# HIV viral load suppression among people with mental disorders at two urban HIV clinics in Uganda: a parallel convergent mixed methods study using the social ecological model

**DOI:** 10.1186/s12981-023-00567-3

**Published:** 2023-09-19

**Authors:** Regina Ndagire, Rachel Nante Wangi, Kevin Ouma Ojiambo, Joanita Nangendo, Juliet Nakku, Herbert Muyinda, Fred C. Semitala

**Affiliations:** 1 Clinical Epidemiology Unit, Makerere University College of Health Sciences, Kampala, Uganda; 2 Butabika National Mental Referral Hospital, Kampala, Uganda; 3 Child Health Development Center (CHDC), Kampala, Uganda; 4 Department of Internal Medicine, Makerere University College of Health Sciences, Kampala, Uganda; 5 Makerere University Joint AIDS Program (MJAP), Makerere University, Kampala, Uganda; 6 Clarke International University, Kampala, Uganda

**Keywords:** HIV, Viral load suppression, Mental disorders, Antiretroviral therapy

## Abstract

**Background:**

Uganda adopted and implemented the Universal Test and Treat (UTT) guidelines in 2017, which require HIV-infected persons to be initiated on antiretroviral therapy (ART) at any CD4 + cell count, and to be routinely monitored for viral load to assess response to ART. However, there is paucity of data on viral load suppression (VLS) among people living with HIV (PLHIV) with mental disorders. We conducted a parallel convergent mixed methods study to determine HIV VLS among people with a mental disorder and explored the socio-cultural determinants of VLS at Butabika hospital and Mulago (ISS) HIV Clinics in Uganda.

**Methods:**

We conducted a retrospective medical records review; seven key informant interviews (KII) among purposively selected healthcare providers and 12 in-depth interviews (IDI) among clinically stable PLHIV with a mental disorder. Data was collected on demographics, mental disorder, ART, viral load status, social support, stigma, and disclosure of HIV status. Quantitative data was analysed using descriptive statistics and modified Poisson regression, while Inductive thematic analysis was used for the qualitative data.

**Results:**

Of the 240 PLHIV with a mental disorder who were enrolled, 161 (67.1%) were female with mean age 38.9 (± 11.2) years. Overall, 88.8% (95% Cl: 84.0 – 92.2%) achieved VLS. Age (aPR = 1.00, 95%Cl = 1.00–1.00), male gender (aPR = 0.90, 95%Cl = 0.82–0.98), divorced (aPR = 0.88, 95%Cl = 0.82–0.94), widowed (aPR = 0.84, 95%Cl = 0.83–0.86), baseline CD4 count < 200 (aPR = 0.89, 95%Cl = 0.85–0.94), psychotic mental disorders (aPR = 1.11; 95%CI = 1.08–1.13) and fair (85–94%) ART adherence level (aPR = 0.69, 95%Cl = 0.55–0.87) and TDF/3TC/DTG (aPR = 0.92; 95%CI = 0.91–0.94) were associated with HIV VLS. Social support from family members, knowledge of impact of negative thoughts on VLS, fear of breaking up with partners and compassionate healthcare providers positively influenced VLS. Stigma and discrimination from the community, self-perceived stigma hindering social relations, socio-economic challenges and psychiatric drug stock-outs negatively affected VLS.

**Conclusion and recommendations:**

HIV VLS among PLHIV with mental disorders at institutions that provide integrated HIV and mental health care is still below the UNAIDS 95% target. Health promotion messaging focusing on benefits of VLS and countering stigma to create a safe environment; and active involvement of family members in care could improve HIV treatment outcomes for PLHIV with mental disorders.

## Introduction

The prevalence of HIV among people with a mental disorder is higher compared to the general population [1–3]. In Uganda, the HIV prevalence among mental health patients is 11.3% [4] compared to 5.8% among adults in the general population [5]. Uganda adopted and rolled-out the Universal Test and Treat (UTT) guidelines in 2017, recommending provision of lifelong antiretroviral therapy (ART) to people living with HIV (PLHIV) irrespective of CD4 or World Health Organization (WHO) HIV clinical stage [6]. However, PLHIV with co-morbid mental disorder may delay ART initiation or not be retained in care [7, 8], suffer poor adherence [9], poor quality of life and increased mortality [10, 11]. These may affect their ability to achieve viral suppression.

Earlier research reported viral non-suppression among PLHIV with major depressive disorder at 12.2–13.5% [12, 13]. However, elsewhere viral load suppression (VLS) among psychiatric patients with various disorders has been reported to be as low as 52% [14]. Mental disorders affect patients’ behavioural functions making it difficult to adhere to ART, attend hospital visits [9] or avoid risky sexual behaviours. Some mental disorders also render patients socially unacceptable and hence stigmatised based on stereotypes tagged to mental illnesses.

Different factors are associated with VLS including age, marital status, treatment adherence, WHO clinical stage [15–19], and some common mental disorders [20, 21]. Socio-cultural factors such as stigma, social support, substance abuse, individual perceptions, and HIV-serostatus disclosure [18, 19, 22–25] also influence VLS. However, these factors have been studied in the general population and remain less understood among PLHIV with mental disorders. This study sought to determine HIV VLS and the associated factors among PLHIV with mental disorders, and explored the socio-cultural factors influencing VLS.

## Materials and methods

### Study design

This was a parallel convergent mixed methods study where quantitative and qualitative data were collected and analysed simultaneously. The findings were interpreted together. The quantitative section used a cross sectional design while the qualitative section employed an exploratory design. The qualitative study was conducted and the results presented following the Social Ecological Model (SEM). The model takes into consideration the individual, and their relations to people, organizations, and community where they reside. It therefore depicts five levels – Individual, Interpersonal, Organizational, Community, and Public Policy [26]. The qualitative design helped us to answer the socio-cultural factors which we wouldn’t get from the quantitative data since the quantitative was obtained through review of patients’ records.

### Study setting

The study was conducted at Butabika National Referral Mental Hospital and Mulago ISS Clinics. Butabika is a national mental health referral and teaching hospital that offers general and specialized mental health treatment. The hospital runs a busy outpatient department which includes the HIV Clinic. The HIV clinic serves about 350 active patients who include PLHIV with and without mental disorders. An estimated 5–15 PLHIV diagnosed with mental disorders attend the clinic every week. Mulago ISS Clinic is also teaching facility located within Mulago National Referral Hospital complex. The clinic provides care to over 17,000 active PLHIV. An estimated 15–20 PLHIV diagnosed with mental disorders attend the clinic every week. Both HIV clinics (at Butabika and Mulago hospitals) are supported by the Makerere University Joint AIDS Program (MJAP), a President’s Emergency Plan for AIDS Relief (PEPFAR) implementing partner in Uganda.

### Quantitative study

#### Study population and sampling

**Fig. 1:**
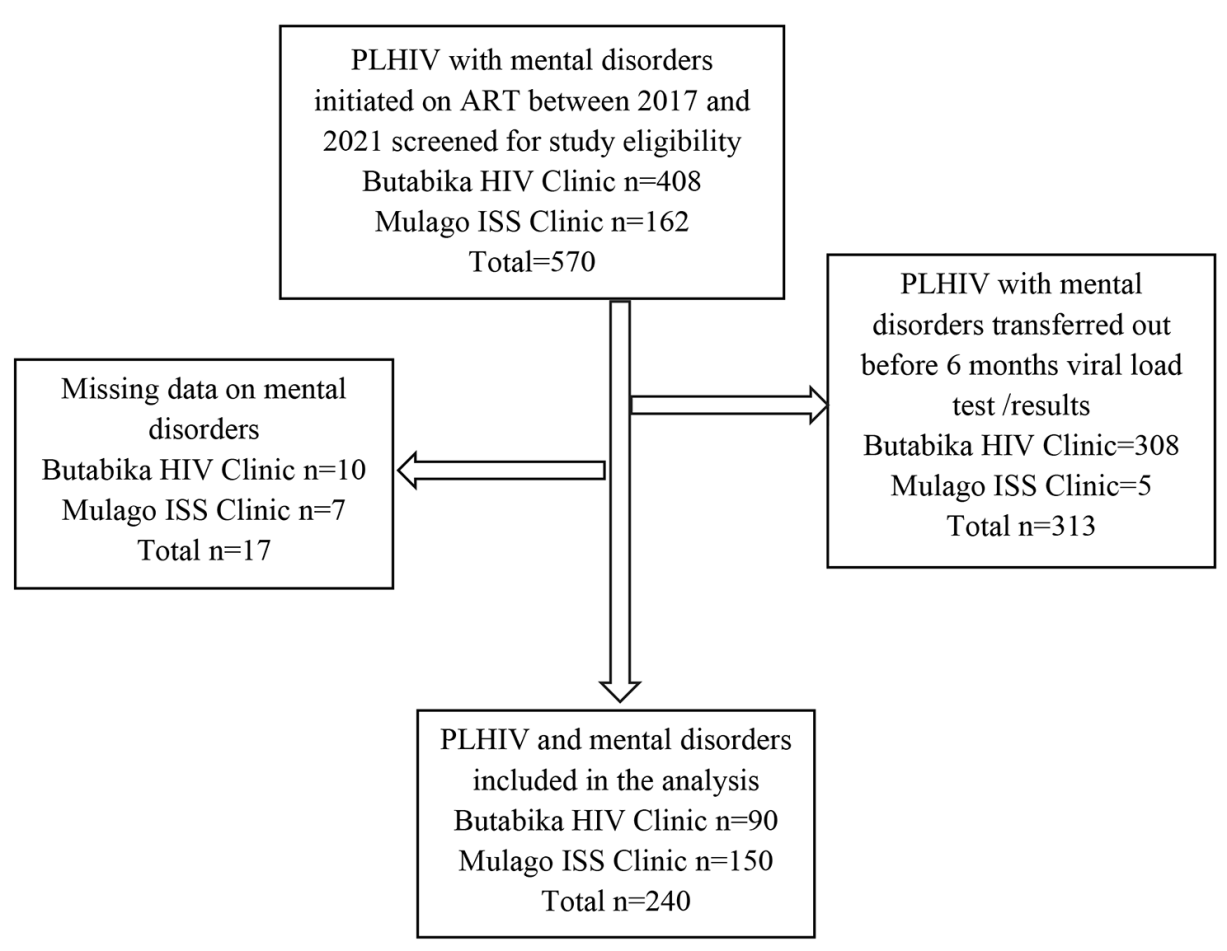
Study profile showing study population selection of the PLHIV with mental disorders at Butabika hospital ART Clinic and Mulago ISS Clinic

PLHIV and mental disorders were eligible if they had: (a) been initiated on ART at Butabika hospital ART clinic or Mulago ISS Clinic between 2017 and 2021; (b) been on ART for at least 6 months; (c) a record of HIV diagnosis; (d) a record of any of the psychiatric diagnoses as per the fifth edition of the Diagnostic and Statistical Manual of Mental Disorders (DSM V) [27] or a clinic record of a history of a mental disorder with atleast 1 filled prescription for any of antipsychotic, anxiolytic, or antidepressant psychiatric medications [28, 29]; and (e) a record of at least one viral load test result after initiation on ART. The study considered the most recent viral load record at a time/visit when the patient was on psychiatric medication [28] for participants that had several results. We purposively selected the year 2017 because it was the year when Uganda rolled out the UTT policy [30]. Sample size was calculated using Keish-Leslie’s formula of sample size estimation. Using Zα corresponding to 95% level of confidence (z) of 1.96, proportion of VLS (p) of 52% [14], level of precision (d) = 0.05 and a design effect of 2 to cater for clustering at clinic level, a sample size of 770 was generated. However, since the population size was limited, we scaled down the study sample size using the formula, $S = N/{1+(N/population{\,}size)}$ where, N is the calculated sample size (770), using an estimated population size = 300 (Butabika – 100; Mulago – 200) according to the respective records officers and S is the adjusted sample size. The final sample size came up to 238 after adjusting for 10% missing data. However, we enrolled all participants at the clinics who met the study eligibility criteria and whose medical records were retrievable. The flow of study participants is shown in Fig. 1.

#### Data collection

Data was collected through document review using a structured data abstraction tool developed from literature. The tool was pilot tested on 10 randomly selected records of PLHIV with a mental disorder who were initiated on ART at Butabika ART clinic in 2016 and revised to address study objectives. Data was abstracted in duplicate by two research assistants, from patients’ medical records. Data was collected on demographics, mental disorder, ART regimen, most recent VL, CD4 cell count, adherence level, WHO Clinical stage, history of TB and psychiatric treatment. We retrieved the adherence level at ART initiation which had been previously determined by self-report or pill count and sub-categorised into poor (< 85), Average (85–94%) and Good (>/= 95) [30]. Discrepancies were resolved through discussion between the research assistants and a third person. The research assistants were trained on study procedures at the beginning of the study. The two research assistants were a registered nurse and clinical officer, and were not involved with routine patient care at the study sites.

### Statistical analysis

Data were analysed using STATA 14 (StataCorp, College Station, TX, USA). Means (and standard deviations) were used to summarize normally distributed numerical variables; and medians (and interquartile ranges) reported for non-normally distributed data. Frequencies and percentages were used to summarize categorical variables. Viral load (VL) < 1000copies/ml was used as the cut off for VLS, the outcome of the study [30]. We used modified Poisson regression with robust standard errors to assess for factors independently associated with VLS. However, because the design effect of the study was found to be one, we did not adjust for clustering in the final analysis.The regression models reported prevalence ratios as the measure of association and their corresponding 95%CI. The study used a 5% level of significance and 95% level of confidence. Independent variables included socio-demographics, and clinical factors. Factors with a p-value < 0.2 at bivariate analysis were considered for multivariable analysis. We assessed for interaction using a chunk test; and confounding using a 10% change in prevalence ratios between the crude and adjusted models. Predictors with p < 0.05 at multivariable analysis were considered significant.

### Qualitative study

#### Population and sampling

Healthcare providers were eligible for KII if they worked at the study sites for at least 12 months and gave written informed consent. Participants for the KII included medical doctors, psychiatric nurses, peer educators, HIV/ART counsellors, and general nurses. The IDI included only clinically stable PLHIV and any mental disorder who fulfilled the criteria for the quantitative survey and were able to give written informed consent. For this study, participants were considered as clinically stable if they were on mental health treatment, not currently admitted at the hospital and were able to bring themselves for their clinic visit. Participants were selected purposively. The adequate sample size was based on data saturation.

#### Data collection

Data was collected through KII and IDI using semi-structured interview guides. Participants for in-depth interviews were identified from records and contacted to participate in the study. They were a subset of the quantitative survey. The interviews were conducted through one-time face-to-face meetings in either English or Luganda depending on the preference of the participants. The interviews were audiotaped and short notes taken by the research assistant during interviews.

#### Data analysis

Data was analysed following an inductive thematic approach. The qualitative section was designed, conducted and findings reported as per the consolidated criteria for reporting qualitative research (COREQ) guidelines [31]. Analysis was done using Open Code software [32] where the coding process was done to generate themes. Two people independently read the transcripts to identify preliminary codes and they subsequently discussed throughout the coding process to resolve discrepancies in their codes until a final codebook was generated. The codes were categorised into sub-themes and mapped in the domains of the social ecological model. Similar codes were merged into one theme; representative of socio-cultural factors that participants reported to influence viral suppression. The results (codebook, sub-themes and themes) were verified by a third person before they were adopted as the final results. The findings were presented with supporting verbatim quotes from the interviews.

## Results

### Quantitative results

#### Study population characteristics

Between November 2021 and January 2022, medical records of 240 PLHIV who had a mental disorder 67.1% of whom were female, mean age of 38.9 (± 11.2) years were reviewed and enrolled into the study (Table 1). Overall, median CD4 cell count was 292 cells per mm^3^ (IQR 131, 457). 40% of participants were in WHO stage III. Nearly three quarters 171 (71.3%) of all participants were on tenofovir/lamivudine/efavirenz (TDF/3TC/EFV) as the initial ART regimen. Majority 98 (47.3%) of the participants had depression while 75 (36.2%) had psychosis as showed in Table 1.

**Table 1:** Sociodemographic and clinical characteristics of the PLHIV with mental disorders at Butabika hospital ART Clinic and Mulago ISS Clinic (n = 240)

Characteristic		Frequency (%)
**Mean age in years (SD)**	38.9 (11.2)	
**Baseline CD4 in cells per mm3** Median (IQR)	292 (131, 457)	
**ART duration in years** Median (IQR)	4.3 (3.6, 4.7)	
**Mental health treatment duration in years** Median (IQR)	4.0 (3.3, 4.5)	
**Gender**		
Female		161 (67.1)
Male		79 (32.9)
**Religion**		
Christian		184 (79.3)
Muslim		48 (20.7)
Missing		8 (3.3)
**Marital status**		
Married		75 (35.90
Single		74 (35.4)
Divorced		49 (23.4)
Widow		11 (5.26)
Missing		31 (12.9)
**Have treatment supporter**		
Yes		215 (89.6)
No		25 (10.4)
**WHO stage**		
Stage III		96 (40.0)
Stage I		39 (16.3)
Stage II		93 (38.8)
Stage IV		12 (5.0)
**Baseline ART regimen**		
TDF/3TC/EFV		171 (71.3)
AZT/3TC/NVP		16 (6.7)
TDF/3TC/NVP		14 (5.8)
TDF/3TC/DTG		26 (10.8)
Other		13 (5.4)
**Adherence level**		
Good>/=95%		202 (84.2)
Fair 85–94%		26 (10.8)
Poor < 85%		12 (5.0
**Mental Disorder**		
**Non-psychotic**		**127 61.4)**
Alcohol and drug use		7 (3.4)
Bipolar		16 (7.7)
Depression		98 (47.3)
Anxiety		6 (2.9)
**Psychotic**		**80 (38.6)**
Psychosis		75 (36.2)
Schizophrenia		5 (2.4)
Missing		33 (13.8)
**History of TB**		
No		212 (88.3)
Yes		28 (11.7)

SD – Standard deviation; IQR – Interquartile range

Missing – #of participants missing data.

#### HIV viral suppression

The overall proportion of HIV VLS was 88.8% (95%Cl: 84.0–92.2). Ninety percent, (95%Cl: 81.8–94.8) in Butabika and 88% (95%Cl: 81.7–92.3) in Mulago. Among PLHIV and mental disorders, male participants were less likely to achieve VLS (aPR = 0.90; 95%Cl = 0.82–0.98). Age had no effect on VLS (aPR = 1.00; 95%Cl = 1.00–1.00). PLHIV with a mental disorder who were divorced (aPR = 0.88; 95%CI = 0.82–0.94) or widowed (aPR = 0.84; 95%CI = 0.83–0.86) were less likely to achieve VLS than the married. Similarly, viral suppression was uncommon among participants with a baseline CD4 count < 200 (aPR = 0.89; 95%CI = 0.85–0.94). Participants who had fair (85–94%) adherence level to ART were less likely to be suppressed compared to those with good (≥ 95%) adherence (aPR = 0.69; 95%CI = 0.55–0.87). Participants on TDF/3TC/DTG (aPR = 0.92; 95%CI = 0.91–0.94) as baseline ART regimen were less likely to be suppressed compared to those on TDF/3TC/EFV. Participants with psychotic mental disorders (aPR = 1.11; 95%CI = 1.08–1.13) were more likely to achieve VLS than those with non-psychotic disorders. The results are summarized in Table 2.

**Table 2:** Demographic and clinical characteristics associated with viral load suppression among PLHIV with mental disorders at Butabika hospital ART Clinic and Mulago ISS Clinic

**Characteristic**	VLS		Crude PR (95%Cl)	p-value	Adjusted PR (95%Cl)	p-value
	Yes **n (%)**	No **n (%)**				
**Mean age in years**			1.00 (0.99–1.00)	**0.189**	1.00 (1.00–1.00)	**< 0.001**
**Gender**						
Female	147 (91.3)	14 (8.7)	1		1	
Male	66 (83.5)	13 (16.5)	0.92 (0.83–1.00)	**0.072**	0.90 (0.82–0.98)	**0.047**
**Religion**						
Christian	164 (89.1)	20 (10.9)	1	1		
Muslim	41 (85.4)	7 (14.6)	0.96 (0.94–0.98)	**< 0.001**	-	**-**
Missing	8 (100)	0 (0)	1.12 (1.11–1.13)	**< 0.001**	-	-
**Marital status**						
Married	67 (89.3)	8 (10.7)	1	1	1	
Single	68 (91.9)	6 (8.1)	1.02 (0.99–1.06)	**0.061**	1.06 (0.91–1.23)	0.453
Divorced	42 (85.7)	7 (14.3)	0.99 (0.89–1.03)	0.229	0.88 (0.82–0.94)	**< 0.001**
Widow	2 (18.2)	9 (81.8)	0.81 (0.86–0.98)	**0.008**	0.84 (0.83–0.86)	**< 0.001**
Missing	27 (87.1)	4 (12.9)	0.97 (0.96–0.99)	**< 0.001**	0.99 (0.93–1.05)	0.728
**Have a treatment supporter**						
Yes	190 (88.4)	25 (11.6)	1		-	-
No	23 (92.0)	2 (8.0)	1.04 (0.86–1.26)	0.677	-	-
**WHO stage**						
Early stage (I&II)	120 (90.9)	12 (9.1)	1		1	
Advanced stage (III&IV)	93 (86.1)	15 (13.9)	0.95 (0.93–0.97)	**< 0.001**	0.94 (0.86–1.03)	0.188
**Baseline ART regimen**						
TDF/3TC/EFV	155 (90.6)	16 (9.4)	1		1	
AZT/3TC/NVP	14 (87.5)	2 (12.5)	0.97 (0.79–1.17)	0.724	0.93 (0.75–1.15)	0.498
TDF/3TC/NVP	12 (85.7)	2 (14.3)	0.95 (0.76–1.18)	0.618	0.86 (0.59–1.25)	0.435
TDF/3TC/DTG	22 (84.6)	4 (15.4)	0.93 (0.87–1.00)	**0.050**	0.92 (0.91–0.94)	**< 0.001**
Other	10 (76.9)	3 (23.1)	0.85 (0.72–1.00)	**0.054**	0.82 (0.71–0.95)	**0.009**
**Adherence level**						
Good > = 95%	188 (93.1)	14 (6.9)	1		1	
Fair 85–94%	17 (65.4)	9 (34.6)	0.70 (0.49–0.99)	**0.049**	0.69 (0.55–0.87)	**0.002**
Poor < 85%	8 (66.7)	4 (33.3)	0.72 (0.59–0.86)	**< 0.001**	0.77 (0.49–1.21)	0.255
**Mental Disorder**						
Non-psychotic	110 (86.6)	17 (13.4)	1		1	
Psychotic	72 (90.0)	8 (10.0)	1.04 (0.93–1.16)	0.502	1.11 (1.08–1.13)	**< 0.001**
Missing	31 (93.4)	2 (6.1)	1.08 (1.05–1.12)	**< 0.001**	1.06 (1.06–1.07)	**< 0.001**
**Baseline CD4**						
≥ 200	148 (91.9)	13 (8.1)	1		1	
< 200	65 (82.3)	14 (17.7)	0.89 (0.82–0.97)	**0.009**	0.89 (0.85–0.94)	**< 0.001**
**History of TB**						
No	187 (88.2)	25 (11.8)	1			
Yes	26 (92.8)	2 (7.1)	1.05 (0.94–1.17)	0.372	-	**-**
**ART duration in years**			1.03 (0.98–1.08)	0.307	-	**-**
**Mental health treatment duration in years**			1.02 (0.99–1.04)	**0.196**	-	**-**

PR – Prevalence Ratio; Cl – Confidence Interval

**Table 3:** Characteristics of 12 PLHIV with mental disorders at Butabika hospital ART Clinic and Mulago ISS Clinic for in-depth interviews

Characteristic	Frequency (%)
**Mean age in years (SD)**	37.9 (9.5)
**Sex**	
Male	4 (33.3)
Female	8 (66.7)
**Viral suppression status**	
Yes	9 (75)
No	3 (25)
**Type of Mental disorder**	
Depression	6 (50)
Psychosis	3 (25)
Alcohol and drug use	1 (8.3)
Anxiety	2 (16.7)
**Marital status**	
Married	5 (41.7)
Single	2(16.7)
Divorced	5 (41.7)
**HIV Clinic**	
Butabika	7 (58.3)
Mulago	5 (41.7)

### Qualitative results

#### Participant characteristics

PLHIV who had a mental disorder (n = 12) and healthcare providers (n = 7) participated in the study for IDI and KII respectively. The summary of characteristics of the participants included for the IDI is presented in Table 3.

### Integration of qualitative and quantitative studies

In the qualitative findings below, we describe the socio-cultural factors that influence HIV VLS according to patients and healthcare providers. We present these factors in relation to the domains of the social ecological model (SEM) [26] which include individual, interpersonal, community, organisational and policy levels. The socio-cultural factors explain the VLS reported in the quantitative study but also put in context other factors that are associated with VLS that were identified from the quantitative survey. The themes that emerged on socio-cultural factors are presented with supportive verbatim quotes below. IDI participants were taken from the quantitative survey sample.

### Socio-cultural factors that influence HIV viral suppression

#### Interpersonal / community domain

Social support systems played a crucial role in enabling VLS. Participants reported that disclosing their HIV status to their immediate and close family members including partners helped them in achieving VLS because of the support that families rendered to them. And for most of the participants, families were aware of their mental health conditions.

“*Both of my parents died but I told my brother [about my HIV status] and he counselled me and escorted me to hospital.*” (**ID 9; suppressed**).

*“I told him [Husband] when I went back home with the [HIV positive] results … he also tested positive. When am busy, he comes [for the drugs] or vice-versa.”* (**ID 10, suppressed**).

*“My mother knows [about HIV and mental disorder] she brings me to hospital sometimes… sometimes she gives me transport or buys the mental health drugs”* (**ID 8; non-suppressed**).

However, a participant in a new relationship expressed fear to disclose her status to her boyfriend because she was afraid of losing him. She, however, indicated that they do not stay together. Although it did not affect the way she takes her medication, non-disclosure makes partners vulnerable to HIV.

“*I have not yet told my boyfriend about my status …but we don’t stay together so am able to swallow my drugs without his knowledge…we have not engaged in sexual activity.*” (**ID 5, suppressed**).

Community-based models of health care delivery may put PLHIV and mental disorders at risk of stigma from the community, which consequently affects their viral load.

“*They [healthcare providers] came in a vehicle to our home and also went to another man’s home who had disclosed his [HIV] status to the community and he does community peer education. So now people also suspect me to have HIV…they keep avoiding me.”* (**ID 4, non-suppressed**).

Participants reported experiencing stigma or discrimination from the community due to either HIV or mental disorder. They noted that although it didn’t stop them from taking their medication, it affected them psychologically. Participants however expressed understanding of the impact that their mental health state could have on their viral load as evidenced by their explanations that negative thoughts were associated with high viral loads.

*“…my sister keeps laughing at me and abusing me…but I take my medicines but of course it tortures me. I have no peace of mind at home.”***(ID 7, suppressed)**.

“*She [Wife] left me, went with the children and stopped them from visiting me…she used to help me with cooking, washing…but I also miss my children…It’s hard to concentrate. The counsellors say you can’t fight the virus if you have negative thoughts but I can’t help it. ”* (**ID 11, suppressed**).

Self-perceived stigma and fear of rejection limited social relations which would otherwise be beneficial. Participants were afraid of building their social networks and this was affirmed by healthcare providers. For some participants, the fear to separate with their partners motivated them to adhere to treatment so as to control their viral load and mental health relapses.

“*Am afraid they will discriminate me …but I also fear making new friends because once they know [the HIV status] they will reject me.”* (**ID 1, suppressed**).

“*I can’t do drugs [abuse drugs] again, she [partner] doesn’t like it and always warns me against relapsing…We swallow the drugs [ARVs] at the same time*.” (**ID 1, suppressed)**.

*“They are afraid of getting partners because of fear of rejection …and worry if they will have children*…*it stresses them and this is not good for them. But partners would support them in taking their medication and be able to control not only HIV but also the mental illness. ”* (**KII 4**).

#### Individual domain

Participants noted to experience some socio-economic challenges that hindered their access to treatment. Majority did not have jobs. They reported to miss hospital visits and treatment because they have no money to buy the drugs that are out of stock at the facility. This negatively impacted VLS.

*“…sometimes I’m unable to come [to hospital] because of transport. At times I have to buy drugs because they run out of stock at the clinic….especially psychiatric drugs. And yet I don’t have money.*” (**ID 6, Suppressed**).

“*I was laid off work because of regular absence due to sickness or hospital visits…my mother has to give me transport to hospital and other personal effects. Yet she can’t afford….*” (**ID 8, non-suppressed**).

#### Community and organizational domain

Supportive patient-provider relationship also positively influenced suppression. Participants reported healthcare providers to be very compassionate during the course of treatment which they believe was key in helping them suppress. However, they also noted some negative experiences with healthcare providers especially due to non-adherence.

“*The counsellors are good. They thank you for swallowing drugs well when the virus reduces. It’s encouraging…sometimes they are harsh like when the virus is not reducing… ”(***ID 12, non-suppressed**).

“*The clinic works on specific days but for these patients [PLHIV with mental disorders] we work on them any day they come*.” (**KII 1**).

Stocking HIV Clinics with mental health drugs and minimising stock-outs would reduce on the waiting time and improve suppression. Participants reported inability to afford to buy mental health drugs that are out-of-stock. This interferes with adherence and may affect viral suppression.

*“…it would be good if the HIV clinic also had mental health drugs. Patients get tired lining up…the drugs run out stock and you have to tell the patient to buy. Most can’t afford and so they miss doses.”*(**KII 4).**

Healthcare providers reported that they emphasise the need for patients to have treatment supporters to help them during the course of treatment which enables them to achieve VLS. Similarly, three quarters of participants in the quantitative survey had a treatment supporter. Treatment supporters included a relative, spouses, friend or fellow patient.

“*It is almost like a requirement to have a treatment supporter. Those that don’t have we encourage them to get treatment buddies. Because they are beneficial; they remind them to take their medication, hospital appointments, and offer physical and psychological support …they leave their [treatment supporter] contacts with the facility*.” (**KII 6**).

More at the institutional level, health care providers reported caring for in-patients but emphasised the need for family involvement in the treatment process. They argued that regular visits from family members motivates patients to adhere to treatment, which improves their viral load.

“…*the nurses on duty help them [in-patients] take their medication. They prioritise PLHIV and mental disorders.”* (**KII 4**) “*…the nurses alone are not enough to provide all the necessary care. Caretakers are encouraged to visit but some [caretakers] dump patients and never visit them*. *…those patients keep relapsing… and remain unsuppressed.*” **(KII 5)**.

## Discussion

This study aimed to determine HIV VLS among PLHIV with a mental disorder and to explore the socio-cultural factors associated with HIV VLS using the social ecological model. We found viral suppression at 88.8% with divorced people, those with a low CD4 cell count and people with poor adherence and those on TDF/3TC/DTG regimen being less likely have VLS. People with psychotic mental disorders were more likely to achieve VLS. Social support from family members, knowledge of impact of negative thoughts on VLS, fear of breaking up with partners and compassionate healthcare providers positively influenced VLS. Stigma and discrimination from the community, self-perceived stigma hindering social relations, socio-economic challenges and psychiatric drug stock-outs negatively influenced VLS.

The overall HIV VLS was 88.8% which is lower than the UNAIDS target of 95% [33]. It is also lower than the VLS in the general population at Mulago clinic (97%) [34] and Butabika clinic (98%) as showed by hospital records. This lower VLS could be due to the challenges attributable to the mental illnesses like non-adherence, poor retention in care as reported in the qualitative findings. However, the results are consistent with findings from northern Uganda [12, 13]. The similarity could be attributable to both studies having been conducted within hospital settings, although the previous studies were done among PLHIV and major depressive disorder only. On the contrary, the HIV VLS in this current study is higher than reported in some studies of similar populations [14, 35]. The disparity could be because of the difference in cut off measures of VLS used. This current study used a higher cut off of < 1000 copies/ml [30].

Among PLHIV and mental disorders, male gender, low baseline CD4 cell count, being divorced or widowed, and poor adherence were associated with low viral suppression. These findings are consistent with findings from studies in sub-Saharan Africa [18, 19, 36, 37]. Consistent with some Ugandan literature, in this study, we found age to have no effect on VLS [38, 39]. Although some studies in America reported VLS to increase with age [15, 28]. Contrary to previous studies, this study found patients on DTG-based regimen to be less likely to suppress compared to patients on EFV-based regimen [40, 41]. This discrepancy could be because the current study did not do real time VL tests but rather reviewed from records which could have introduced bias. We found people with psychotic disorders to be more likely to achieve suppression than those with non-psychotic disorders, contrary to literature [21, 28, 42]. The disparity maybe explained by differences in measures of mental disorders used in the studies. The current study was limited by relying on only medical records with no real time screening to ascertain mental disorders, which previous studies did. Additionally, the participants in the current study could have been stable on treatment and hence not entirely reflective of the situation in the community.

At the interpersonal level of the socio-ecological model, participants reported disclosing their HIV status to immediate family as encouraged by healthcare providers but were reluctant to disclose to community members. This earned them social support which they say helped them suppress the virus. Healthcare providers noted that relatives supported patients in treatment if they knew about their conditions which helped in achieving viral suppression. The support rendered included doing their laundry, preparing and providing meals and housing, support in activities of daily living, financial provision, picking for them drugs, escorting them on hospital visits among other. This nature of social support network helped patients to adhere to treatment and achieve suppression as seen in previous studies findings [23]. This is consistent with the quantitative findings where majority of the participants reported to have treatment supporters and could explain the VLS reported in the current study [24]. However, the fear of losing social relations like partners and discrimination could have hindered some participants from disclosing about their HIV status which increases risk of transmission and reinfection [43]. Similarly supportive health service provision also enabled participants to achieve suppression. Participants reported healthcare providers to be compassionate. For some this encouraged them to adhere to treatment hence achieving suppression.

However, stigma and segregation was a common problem among PLHIV and mental disorders. Participants reported experiencing double stigma due to HIV and mental disorders. The commonest reported forms of stigma were public stigma and self-perceived stigma. Self-perceived stigma hindered participants from engaging in meaningful relations due to fear of rejection. The stigma could be due to disclosure since majority had disclosed and could also be the reason why some participants (11.2%) in quantitative analysis were unsuppressed. The findings are consistent with literature [22, 44].

Notably, participants expressed knowledge of the importance of having a positive attitude in achieving good treatment outcomes. They noted that negative thoughts hindered viral suppression as is stated in literature [45]. This could explain the level of suppression we found in the survey.

The other hindrance to suppression at individual level that was reported were socio-economic constraints. Most participants did not have a source of income and hence were unable to fend for themselves. Many depended on their family members for survival. This affected their adherence to psychiatric drugs especially when they were out of stock essentially affecting their viral suppression. Some participants even missed hospital appointments due to lack of transport to facilities. This is consistent with previous research where low relative wealth was associated with lack of viral suppression [46]. Additionally, both patients and healthcare providers acknowledged drug stock outs especially for psychiatric drugs adding that most patients cannot afford to buy these drugs since they are expensive. This caused them to default on psychiatric medication and to get mental relapses and subsequently defaulting on ART. In such scenarios patients have been noted to remain virally non-suppressed [47].

Limitations of this study included information bias as patients who received psychiatric medications for indications not related to mental disorders might have been misclassified. Similarly, those who had mental disorders but remained untreated were left out in this study. To prevent this, the study employed a standardised definition of a mental disorder adopted from previous similar studies [27–29] that helped in minimising misclassification. The in-depth interviews involved patients with mental health issues; although clinically stable, there could have been inconsistences in the information that they provided. However, the interviewer repeated selected questions at different time-points during the interview to ascertain that the participants’ responses were consistent. The study used secondary data which is prone to errors and missing data. About 30 participants were lacking some information on either one or more of marital status, religion, age, baseline CD4, mental health diagnosis or duration on mental health treatment. However, at design stage, the sample size had been adjusted to cater for missing data. The cross sectional design also limited detection of causal associations. We did not find a relationship between having treatment support and viral suppression contrary to previous research. However, we are unaware of previous studies on viral suppression among PLHIV and (any) mental disorder, hence the novelty of this study was its strength.

The findings from this study demonstrate the need for health promotion messaging focusing on the benefits of VLS. This could be drawn from findings at the individual and interpersonal level of the SEM, where PLHIV and mental disorders expressed the desire to have partners, and start families. Messaging that counters stigma in communities would also create a safe environment to improve their viral loads. At the individual level, programs need to incorporate income generating activities since finances were a common challenge among PLHIV and mental disorders. The other lesson from the interpersonal domain points to the role of active involvement of family members in the care for PLHIV and mental disorders in improving viral suppression. Additionally, at the institutional level, there’s need to minimise patient waiting time and provision of adequate mental health drugs for PLHIV and mental disorders. Further research could explore changes in viral suppression over time among PLHIV and mental disorders.

In conclusion, HIV VLS among PLHIV and mental disorders at institutions that provide integrated HIV and mental health care is still below the UNAIDS 95% target. Adherence to ART and low CD4 at ART initiation are associated with viral suppression among PLHIV and mental disorders. Social support from family members, knowledge of impact of negative thoughts on VLS, fear of separation with partners and compassionate health care providers positively influenced VLS. Stigma and discrimination from the community, self-perceived stigma hindering social relations, socio-economic challenges and psychiatric drug stock outs at facilities negatively influenced viral suppression. The qualitative findings offer insights that institutions offering integrated HIV and mental health care can look into to improve treatment outcomes among PLHIV and mental disorders.

## Data Availability

The dataset upon which the conclusions of this study were made, is available at Mendeley Data repository: https://data.mendeley.com/ (10.17632/zz82rwg6zp.1.

## List of abbreviations

ART: Antiretroviral therapy
IDI: In-depth interview
ISS: Immune Suppression Syndrome
KII: Key Informant Interview
MJAP: Makerere Joint AIDS Program
PLHIV: people living with HIV
UNAIDS: Joint United Nations Program on HIV/AIDS
UTT: Universal Test and Treat
VL: viral load
VLS: viral load suppression
